# Specific cytoarchitectureal changes in hippocampal subareas in daDREAM mice

**DOI:** 10.1186/s13041-016-0204-8

**Published:** 2016-02-29

**Authors:** Britt Mellström, Asta Kastanauskaite, Shira Knafo, Paz Gonzalez, Xose M. Dopazo, Ana Ruiz-Nuño, John G. R. Jefferys, Min Zhuo, Tim V. P. Bliss, Jose R. Naranjo, Javier DeFelipe

**Affiliations:** Spanish Network for Biomedical Research in Neurodegenerative Diseases, CIBERNED, Madrid, Spain; National Biotechnology Center. CSIC, Darwin, 3. E-28049, Madrid, Spain; Cajal Institute, CSIC Madrid, Av Dr. Arce,37 E-28006, Madrid, Spain; Biomedical Technology Center, Politecnica University Madrid, Madrid, Spain; Neuronal Networks Group, School of Clinical and Experimental Medicine, University of Birmingham, Birmingham, UK; Department of Physiology, Faculty of Medicine, University of Toronto, 1 King’s College Circle, Toronto, Ontario Canada; Center for Neuron and Disease, Frontier Institute of Science and Technology, Xi’an Jiaotong University, Xi’an, China; MRC National Institutes for Medical Research, Mill Hill, London, UK; Present address: IkerBasque Basque Foundation for Science and BioCruces, Health Research Institute, Bizkaia, Spain

**Keywords:** Calcium, Arc, Dendritic trees, Spines

## Abstract

**Background:**

Transcriptional repressor DREAM (downstream regulatory element antagonist modulator) is a Ca^2+^-binding protein that regulates Ca^2+^ homeostasis through gene regulation and protein-protein interactions. It has been shown that a dominant active form (daDREAM) is implicated in learning-related synaptic plasticity such as LTP and LTD in the hippocampus. Neuronal spines are reported to play important roles in plasticity and memory. However, the possible role of DREAM in spine plasticity has not been reported.

**Results:**

Here we show that potentiating DREAM activity, by overexpressing daDREAM, reduced dendritic basal arborization and spine density in CA1 pyramidal neurons and increased spine density in dendrites in dentate gyrus granule cells. These microanatomical changes are accompanied by significant modifications in the expression of specific genes encoding the cytoskeletal proteins Arc, Formin 1 and Gelsolin in daDREAM hippocampus.

**Conclusions:**

Our results strongly suggest that DREAM plays an important role in structural plasticity in the hippocampus.

**Electronic supplementary material:**

The online version of this article (doi:10.1186/s13041-016-0204-8) contains supplementary material, which is available to authorized users.

## Background

Change in intracellular free calcium concentration has long been recognized as a universal signal underlying neuronal plasticity and adaptive responses in the CNS to different environmental stimuli [1, 2]. Diverse signaling pathways participate in these responses, among them a specific set of proteins that decode the calcium signal in accordance with frequency, subcellular location and intensity [3–5]. Despite extensive investigation, however, a detailed mechanistic description of Ca^2+^-dependent signaling in the expression of the late, transcription-dependent component of LTP and LTD remains elusive [reviewed in 6]. It was proposed that the concentration of intracellular free calcium affects dendritic spine (for simplicity, spine) density by controlling spine growth and pruning [7] and that the formation of new spines requires calcium-dependent CREB phosphorylation and CRE-dependent transcription [8].

DREAM belongs to a group of four highly conserved genes (*K*^*+*^*c*hannel *i*nteracting *p*roteins, KChIP-1 to 4) [9, 10], that regulates synaptic activity through different mechanisms (Table 1) including binding to DRE regulatory sites in target genes and to other nucleoproteins like CREB [11, reviewed in 12]. Despite its potential, the role of the Ca^2+^-dependent transcriptional repressor DREAM in spine growth and remodeling associated with the expression of LTP and LTD has not been analyzed.

**Table 1:** DREAM is a multifunctional regulatory protein

Modified function	Regulated by	Molecular mechanism	References
Kv4 channel gating & membrane localization	Calcium Lipids Phosphorylation by GRK2	Protein-protein interaction	An et al., 2000 [10] Holmqvist et al., 2001 [70] Ruiz-Gomez et al., 2007 [71]
Ca^2+^ release from the ER	Not known	Protein-protein interaction	Lilliehook et al., 2002 [72]
Voltage-gated Ca^2+^ channel expression & gating	Calcium	Transcriptional regulation	Ronkainen et al., 2011 [73] Naranjo & Mellstrom, 2012 [24]
		Protein-protein interaction	Thomsen et al., 2009 [74] Anderson et al., 2010 [75]
Ca^2+^ influx through NMDA receptors	Calcium	Protein-protein interaction	Wu et al., 2010 [14] Zhang et al., 2010 [15]
GABAergic inhibition	Calcium	Transcriptional regulation	Mellstrom et al., 2014 [16]
Chronic pain desensitization	Calcium & BDNF	Transcriptional regulation	Rivera-Arconada et al., 2010 [30]
ATF6 processing	Calcium	Protein-protein interaction	Naranjo et al., 2016 [25]
Pain perception	Calcium	Transcriptional regulation	Carrion et al., 1999 [9] Cheng et al., 2002 [26]
		Protein-protein interaction	Hu et al., 2006 [76]

Mutation of the EF-hands in DREAM results in a Ca^2+^-insensitive repressor that in vitro shuts down DRE- and CRE-dependent transcription in the presence of elevated intracellular levels of free Ca^2+^ [9, 13]. Mutation of a leucine-charged residue rich domain (LCD) at the N-terminal of DREAM (L47,52 V) prevents the interaction with CREB [13] and in combination with the EF-hand mutation generates a calcium insensitive double mutant daDREAM that specifically blocks Ca^2+^-/DREAM-dependent transcription without blocking CREB-dependent gene expression. Use of transgenic mice over expressing the Ca^2+^-insensitive DREAM mutant daDREAM revealed that long-term depression (LTD), a form of synaptic plasticity, was significantly impaired in daDREAM transgenic mice [14]. Moreover, contextual fear and spatial memory as well as behavioral anxiety were significantly impaired in daDREAM mice. A postsynaptic modulation of the NMDA receptor by DREAM through a Ca^2+^-dependent interaction with PSD-95 [14] or by the interaction with the NMDA-R1 subunit [15] could also contribute to this phenotype. In addition, expression of daDREAM in the forebrain resulted in a complex phenotype characterized by loss of recurrent inhibition and enhanced LTP in the dentate gyrus (DG), impaired learning and memory and profound changes in the expression of specific activity-dependent transcription factors in the hippocampus, including Npas4, Nr4a1, Mef2c, JunB and c-Fos [16].

Here, we report specific changes in dendritic arborization and spine density in CA1 pyramidal neurons and granule cells of the DG, respectively, in adult transgenic mice expressing the daDREAM mutant. Moreover, changes in the expression of genes related to the cytoskeleton that could participate in the modified cyto-architecture were found in daDREAM transgenic hippocampus.

## Results

**Fig. 1:**
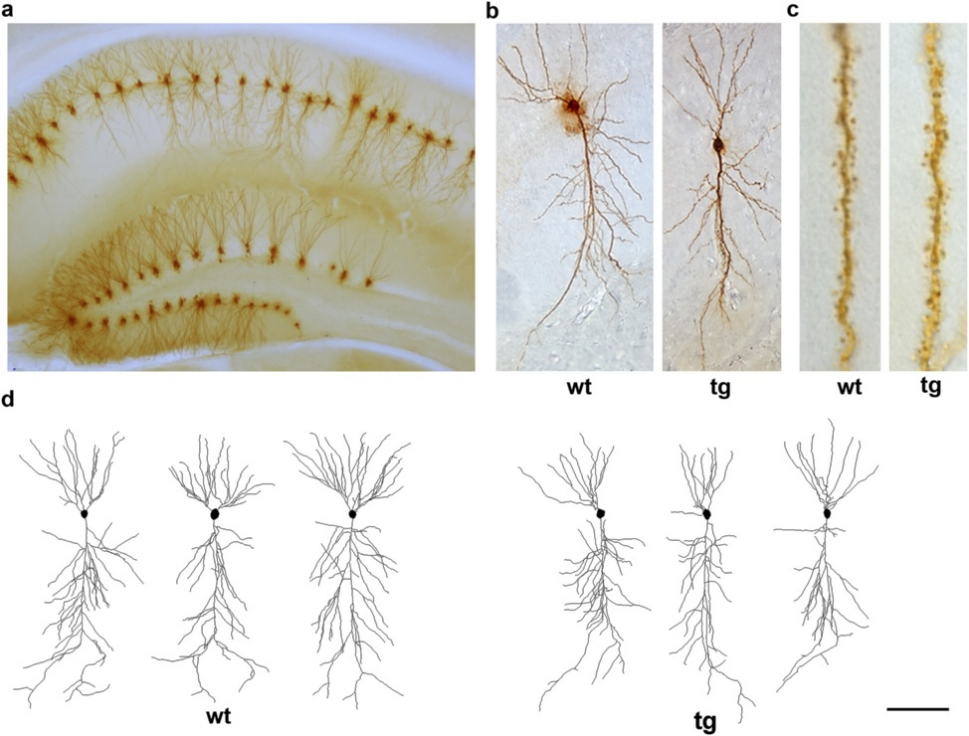
Lucifer Yellow injected neurons in the hippocampus. **a** Panoramic view of the hippocampus showing Lucifer Yellow injected neurons in CA1 and DG areas. **b** Representative individual CA1 pyramidal neurons, wild type (wt) and daDREAM (tg). **c** High magnification photomicrographs of representative dendrites of the CA1 basal dendritic tree, showing the dendritic spines in wt and tg neurons. **d** Neurolucida reconstructions of CA1 wt and tg neurons. Scale bar, A 200 μm, B 40 μm, C 8 μm, D 50 μm

Individual hippocampal neurons were injected with Lucifer Yellow in fixed coronal slices. We could thus readily visualize the dendritic arbor, including fine branches as well as dendritic spines, of individual neurons (Fig. 1a-c). Since in a 200 μm slice the entire dendritic tree could not always be included, due to its large extension, the values regarding the total dendritic lengths and the Sholl analysis for the dendritic tree (length and number of intersections) represent the dendritic tree included in the slice. Nevertheless, as all neurons were injected at the same depth into the slice (30 μm from the surface) it is assumed that the portion of the dendritic tree included in the slice is equal in different neurons and across genotypes.

### Changes in the CA1 dendritic trees in daDREAM mice

Changes in synaptic plasticity and learning and memory are associated with dendritic development [7, 17, 18] as well as with the Ca^2+^-dependent growth and pruning of dendritic spines [19]. Thus, we examined whether neuronal morphology and spine density were altered in daDREAM CA1 pyramidal neurons, those that could be responsible for the modified LTD in daDREAM mice (Fig. 1b and c). Representative tracings of CA1 pyramidal neurons from wild type and daDREAM mice are shown in Fig. 1d.

**Fig. 2:**
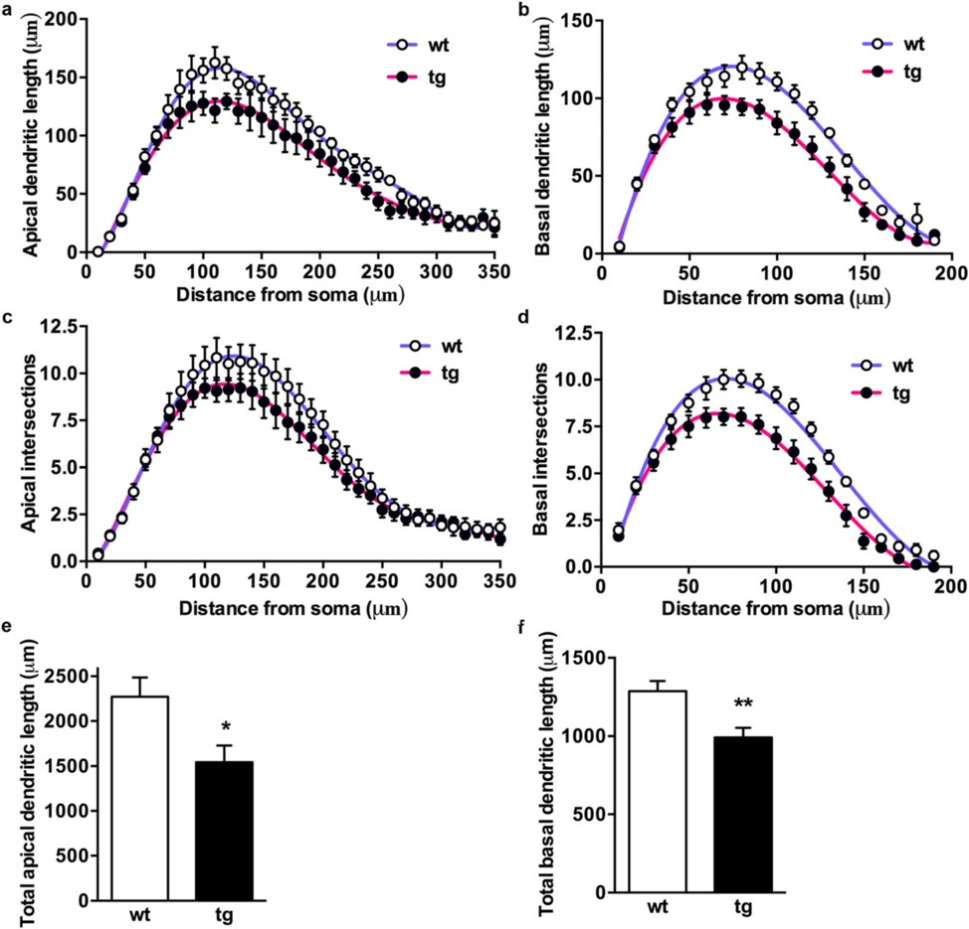
Analysis of the dendritic trees in CA1 of wild type and daDREAM mice. Sholl analysis of the apical (**a**, **b**) and basal (**c**, **d**) dendritic tree showing reduction in length and number of intersections in tg mice (wt, *n* = 5 and tg, *n* = 7; Two-way ANOVA, *P* < 0.0001 for genotype). For **a** and **b** centered sixth order polynomial curve fitting was used (*P* < 0.0001). For **c** and **d** centered third order polynomial curve fitting was used (*P* < 0.0001). Analysis of the total dendritic length revealed smaller apical (**e**) and basal (**f**) total dendritic length in tg mice (wt, *n* = 5, 76 neurons; tg, *n* = 7, 73 neurons; * = 0.042, ** *P* = 0.0052, Mann–Whitney)

Sholl analysis was used to calculate the number of dendrite crossings (intersections) and the dendritic lengths at increasing distances (10 μm interval) from soma as objective measurements of the dendritic complexity. The analysis revealed, both for apical (Fig. 2a, b) and basal (Fig. 2c and d) dendrites, a statistically significant reduction for these parameters in transgenic mice. Thus, the total dendritic tree is shorter (Fig. 2e and f) and the dendritic complexity is smaller in daDREAM CA1 neurons compared to wild type.

**Fig. 3:**
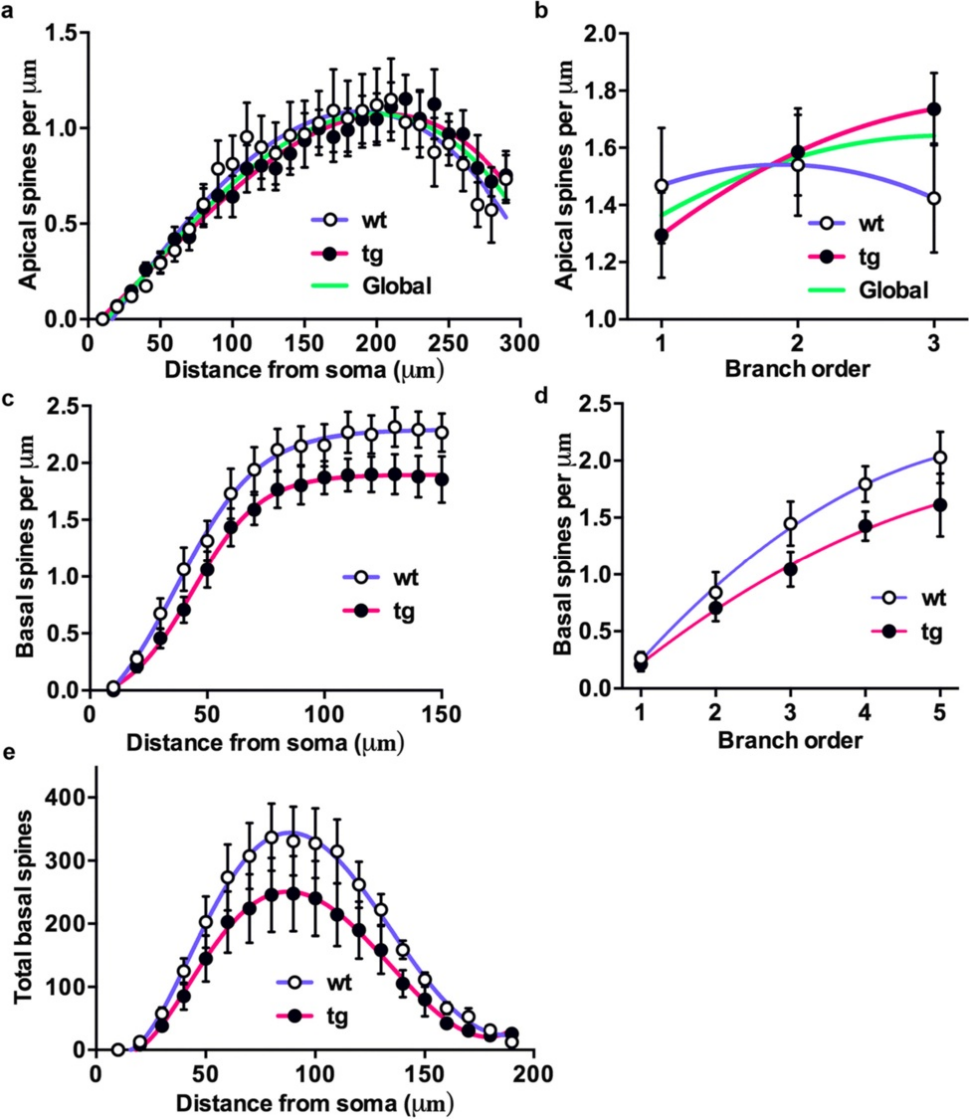
Analysis of spine density in CA1 of wild type and daDREAM mice. Analysis of spine densities in apical and basal dendrites of hippocampal CA1 neurons from wild type and daDREAM mice. **a** and **b** In apical dendrites, no difference between genotypes was observed using Sholl analysis (wt, *n* = 7; tg, *n* = 9). **c** and **d** In basal dendrites, a significant reduction in spine density in tg neurons was observed using Sholl analysis (wt, *n* = 7; tg, *n* = 9). **e** Total number of spines in the basal dendritic tree showing statistically significant lower number in daDREAM neurons (*n* = 8 mice in each group; Two-way ANOVA, P < 0.0011for genotype). Centered sixth order polynomial curve fitting was used (*P* < 0.0006)

Spine density in CA1 neurons was analyzed at increasing distances from soma (Sholl analysis) and for each branch order in apical (Fig. 3a and b) and basal (Fig. 3c and d) dendrites. The result showed no difference in spine density between wt and daDREAM neurons in apical dendrites (Fig. 3a and b). In basal dendrites, however, we found a significant lower spine density in daDREAM neurons (Fig. 3c and d).

In addition we calculated the total number of spines by combining spine density and Sholl analysis (density of spines x dendritic length). We found a significant reduction of spines in the basal arbor of transgenic neurons (Fig. 3e).

### Changes in spine density in DG granular neurons of daDREAM mice

**Fig. 4:**
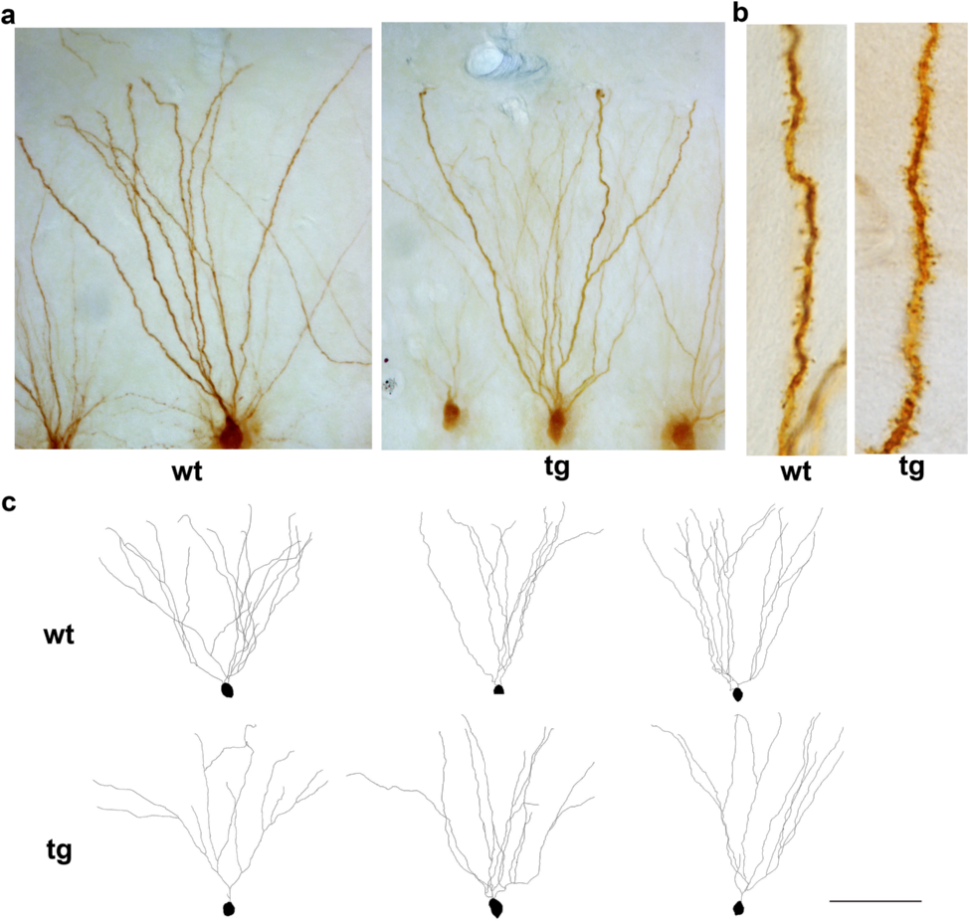
Lucifer Yellow injected DG neurons. **a** Representative DG granular neurons, wild type (wt) and daDREAM (tg). **b** High magnification photomicrographs of representative dendrites of DG wt and tg neurons, showing the dendritic spines. Note the increased spine density in the tg dendrite. **c** Neurolucida reconstructions of wt and tg granular neurons. Scale bar, A 60 μm, B 12 μm, C 100 μm

Individual granular neurons were injected with Lucifer Yellow and traced in three-dimensions (Fig. 1a and Fig. 4a and b). Representative tracings of DG granular neurons from wild type and daDREAM mice are shown in Fig. 4c.

**Fig. 5:**
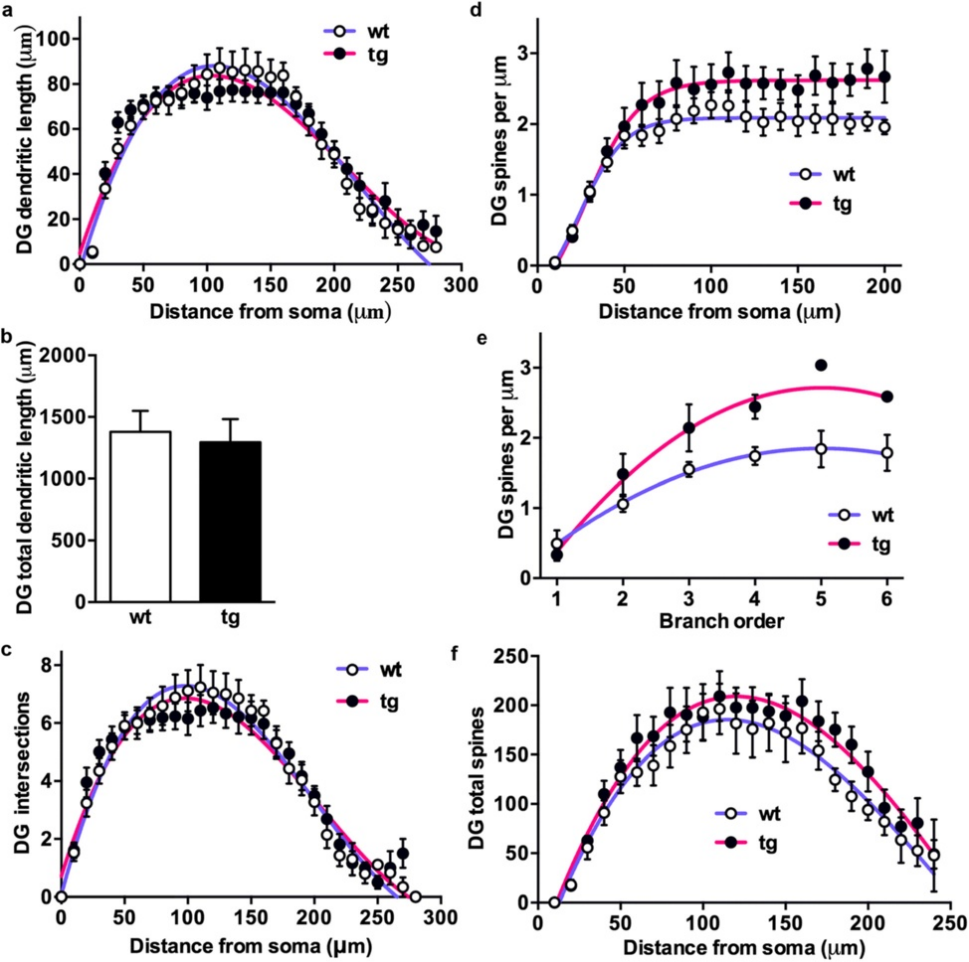
Analysis of spine density in DG neurons of wild type and daDREAM mice. **a** Sholl analysis showing equal dendritic length in wild type (wt) and daDREAM (tg) mice at all distances from soma. **b** The number of the intersections in granular neurons is similar between genotypes at all distances from soma. A significant increase in spine density in tg neurons was observed by Sholl analysis (**c**) (wt, *n* = 7; tg, *n* = 7, Two-way ANOVA, *P* < 0.0001 for genotype) and measured per branch order (**d**) (wt, *n* = 7; tg, *n* = 7, Two-way ANOVA, *P* < 0.001 for genotype). For **c**, sigmoidal curve fitting was used (*P* < 0.0001). For **d**, centered second order polynomial curve fitting was used (*P* < 0.0005). **e** Total number of spines in the basal dendritic tree showing statistically significant higher number in daDREAM neurons (*n* = 8 mice in each group; Two-way ANOVA, *P* < 0.001). Centered third order polynomial curve fitting was used (*P* < 0.0052)

As for CA1 pyramidal neurons, Sholl analysis was used to calculate the number of dendrite crossings (intersections) and the dendritic lengths at increasing distances (10 μm interval) from soma as objective measurements of dendritic complexity. This analysis revealed for daDREAM granule neurons no significant differences in the dendritic length (Fig. 5a and b) and in the number of intersections at any distance from the soma (Fig. 5c).

Spine density, however, was significantly higher in daDREAM compared to wild type granule neurons as measured by Sholl analysis (Fig. 5d) and per branch order (Fig. 5e). As a result, the total number of spines calculated by combining spine density and Sholl analysis (density of spines x dendritic length) was significantly increased in DG transgenic granule cells (Fig. 5f).

### Expression of cytoskeletal related genes are modified in daDREAM hippocampus

**Fig. 6:**
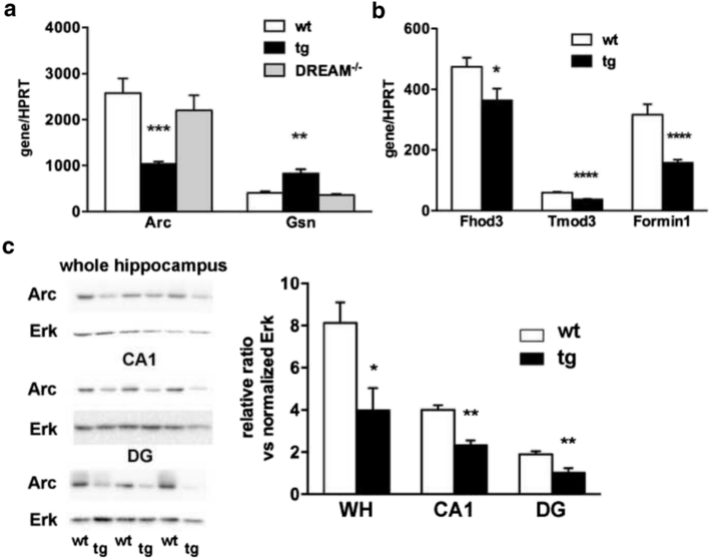
Changes in the expression of cytoskeletal related genes in daDREAM hippocampus. **a**, **b** Quantitative real-time PCR analysis of the indicated genes in hippocampus from wild type (wt), daDREAM (tg) and DREAM knockout (DREAM^−/−^) mice. Values are normalized with respect to HPRT mRNA content. Results are the mean ± SEM. In (a), ** *P* < 0.01, *** *P* < 0.001 (One-way ANOVA followed by Dunnett’s multiple comparison, *n* = 10). In (b), * *P* < 0.05, **** *P* < 0.0001 (Student’s *t*-test, *n* = 17). **c** Western blot analysis of the Arc protein in whole hippocampus (WH), CA1 and DG from wild type (wt) and daDREAM mice (tg). Five mice were analyzed in each group. Representative gels are shown. * *P* < 0.05, ** *P* < 0.01 (Mann Whitney)

To relate morphological changes in transgenic hippocampus with potential differences in gene expression in daDREAM mice, we search for modified levels of mRNAs encoding proteins related to actin polymerization and cytoskeleton, some of which have been related to calcium homeostasis, synaptic plasticity and learning and memory, i.e. Arc [20–22] and Gelsolin [23]. For this, we revisited the results from a genome-wide analysis of daDREAM hippocampus (Gene Expression Omnibus accession number GSE17844) [16] and validated specific messengers by quantitative real-time PCR. Expression levels for *Arc*, *Fhod3*, *Tmod3* and *formin1* were reduced, while a significant increase in *gelsolin* mRNA was observed in daDREAM hippocampus (Fig. 6a and b). These changes were specific since no significant alteration was observed for others, including *formin2*, *spire1* and *cap1* (Additional file 1). Notably, expression of *Arc* and *gelsolin* was not significantly modified in DREAM^−/−^ hippocampus, a lack of effect likely due to compensation by other KChIPs expressed in this brain area (Fig. 6a).

Reduced expression of Arc in whole hippocampus was observed also at the protein level in daDREAM mice (Fig. 6c). Furthermore, analysis of Arc protein in hippocampal subareas showed a decrease in Arc content in the CA1 and the DG from transgenic mice compared to wild-type (Fig. 6c).

Taken together, these results indicate that daDREAM-induced changes in the expression levels of genes related to actin polymerization and to the cytoskeleton, may underlie changes in neuronal morphology and connectivity and could be related to the alterations in more elaborated functions as learning and memory [16].

## Discussion

DREAM regulates the expression of several genes, which are important for Ca^2+^ and protein homeostasis and synaptic plasticity [24, 25]. This includes activity-dependent expression of c-fos and Npas4 [9, 16], as well as, effector genes directly responsible for synaptic events and calcium homeostasis in the postsynaptic neuron such as prodynorphin, BDNF and the sodium-calcium exchanger 3 [26–29]. In addition, expression of daDREAM in the CNS has been associated with modified LTD and LTP responses and a severe impairment in learning and memory formation [14, 16, 30].

Here, we present evidence for permanent changes in the microanatomy of CA1 pyramidal cells and granule cells of DG, which suggest alterations of hippocampal connectivity, as well as in the expression of cytoskeletal genes, *Arc*, *formin1* and *gelsolin*, which could underlie altered LTD and LTP in daDREAM mice.

Modified protein expression of Arc, Formin1 and Gelsolin has been associated with changes in actin polymerization, spine density, dendritic growth and impairment in long-term forms of synaptic plasticity, including LTP and LTD [22, 31, 32]. Thus, it is well characterized that decreased protein expression of Arc impairs LTD in CA1 neurons [33] and in Purkinje cells [20] and that reduced LTD depends on a decrease in the translation rate of pre-existing levels of *Arc* mRNA in CA1 neurons [34]. Moreover, knockdown of Arc in basolateral amygdala impairs long-term extinction of fear memory [35] and total ablation of Arc in Arc^−/−^ mice results in decreased spine density and altered spine morphology in CA1 and DG neurons [36] while no change in spine density was reported earlier [33].

Our results in daDREAM mice add new pieces of information to this functional scenario. We found no difference in spine density between wild type and daDREAM CA1 neurons in apical dendrites, while in basal dendrites we found a significant lower spine density in daDREAM neurons. This difference could be relevant since the mechanisms of induction and maintenance of LTP differ in apical (stratum radiatum) and basal dendrites (stratum oriens) of hippocampal CA1 pyramidal neurons [37–41]. Furthermore, we found an increase in spine density in the granule cells of the DG that may account for the increase in LTP in daDREAM mice observed in this region. Reduced expression of Arc mRNA in whole daDREAM hippocampus translates to a reduction of Arc protein in whole hippocampus, that is also observed in CA1 and DG hippocampal subareas. Thus, complex changes in hippocampal cytoarchitecture in daDREAM mice are not explained solely by the reduction in Arc protein content and might be understood only in the context of a largely modified transcriptional scenario due to daDREAM overexpression [16]. Future experiments should explore changes in the level and distribution of other cytoscheletal proteins, changes in the fine morphology and functionality of the spines in daDREAM hippocampal neurons and the relationship of these two events with the different mechanisms of induction and maintenance of LTP between apical and basal dendrites.

Decreased *formin1* expression and reduced dendritic complexity in daDREAM CA1 neurons may well be related. It has been reported that Formin1 mediates Neurogenin3-induced dendritogenesis and synaptogenesis in cultured hippocampal neurons [42]. Moreover, the increase in *gelsolin* expression in daDREAM hippocampus may be involved in the increase in spine density in daDREAM granule cells in the DG, since a role in the stabilization of actin polymerization has been proposed for Gelsolin [31]. Nonetheless, expression of *cap1* a regulator of Cofilin, an actin related protein that participates in spine changes during LTD [43], is not modified in daDREAM neurons. Thus, long term structural changes and changes in the expression of cytoskeletal proteins have a relatively good correlation that is not complete, suggesting the involvement of additional mechanisms and/or the occurrence of specific changes within hippocampal regions that should be further explored to fully understand the modified long term synaptic plasticity in daDREAM mice.

Down regulation of *Arc* and *formin1* gene expression is in line with the intrinsic repressor activity of DREAM, while the induction of *gelsolin* expression may be secondary to primary changes in target genes or the consequence of the interaction between DREAM and other nucleoproteins important for *gelsolin* gene regulation. Whether DREAM acts directly on the *Arc* and *formin1* promoters or indirectly affecting the transactivating effect of SRF on the distal enhancer that directs basal and activity-dependent transcription of the *Arc* gene [44–46] remains to be clarified. In addition, the decrease in *Arc* expression could be secondary to the repression of *Npas4* and *BDNF* expression in daDREAM hippocampus [16]. Nevertheless, it has been shown that Formin-mediated actin polymerization is involved in the activation of SRF in the nucleus and that SRF activation results in changes of the expression levels of multiple cytoskeletal-related proteins [47–49]. Whether the down regulation of the *formin1* gene is the primary transcriptional event in daDREAM neurons and whether changes in the expression of *Fhod3*, *Tmod3* and *gelsolin* are related to changes in SRF function are questions that remain to be investigated.

A large dendritic arbor implies that a wider region of the cortex must be sampled, while a more complex branching pattern may determine the degree to which the integration of inputs is compartmentalized within their arbors [50–54]. Greater potential for compartmentalization results in a significant increase in the representational power and a greater capacity for learning and memory [54, reviewed in 55]. In addition, each spine receives at least one excitatory synapse and such spines represent the main target of these synapses [56]. Thus, the differences in the size, branching pattern and spine density of cells result in variation in the total number of putative excitatory inputs sampled by each cell and in the integrative properties of these neurons. The lower number of spines in basal dendrites of CA1 and increased spine density in transgenic granule cells of the DG suggests decreased connectivity in the CA1 and enhanced connectivity in the DG. Nevertheless, we have not analyzed the different types of spines present in excess in daDREAM versus wild type neurons, and more important, we do not know how synapses are modified during LTD and LTP in transgenic hippocampus.

Studies using DREAM knockout mice showed no difference with wild type mice in paired pulse facilitation, resting membrane potential or input–output relation of fEPSPs [57]. Moreover, DREAM deficient mice did not show an obvious phenotype in a place-learning version of the Morris water maze test [26] and had only a slight hormonal dependent changes in memory in fear conditioning tests [58] and slight increase in LTP in the dentate gyrus of the hippocampal formation [57]. In the latter study, enhanced LTP in the DG of DREAM knockout mice could be mimicked by potassium channel blockers and was associated with decreased A-type current density [57]. Therefore, it was concluded that Kv4 potassium channels are important for mediating the function of DREAM in synaptic plasticity. However, work in transgenic spinal cord neurons have shown no change in the activity of Kv4 channels in daDREAM transgenic neurons indicating that the lack of spinal sensitization in transgenic mice is not related to change in K^+^ currents but rather related to the transcriptional control of BDNF by mutated DREAM [30]. In the same way, no significant change in the basal expression of Arc, forming 1 and gelsolin were observed in this study in the hippocampus of DREAM −/− mice. Absence of strong phenotypes in DREAM/KChIP3 deficient mice is likely due to the functional redundancy among DREAM/KChIP proteins and their overlapping expression patterns [27, 59], as occurs after genetic ablation of KChIP2 [60] or KChIP1 [61], in which compensation by other KChIP proteins also results in mild phenotypes.

Activity-dependent synaptic plasticity might transform small and silent synapses to larger and fully functional synapses. Expression of the calcium insensitive DREAM mutant in daDREAM mice indicates that not only basal but also activity-dependent changes in gene expression are reduced in daDREAM neurons. Thus, functional synaptic changes upon membrane depolarization may not occur or be reduced in daDREAM compared with wild type neurons. Future studies should address these different possibilities.

## Conclusions

Our results strongly suggest that DREAM plays an important role in learning-related structural plasticity in the hippocampus.

## Methods

### Transgenic mice

The generation of transgenic mice has been reported previously [29, 30]. This study was performed in daDREAM mice line 26, specific details about this transgenic line can be found in [16].

All experimental protocols involving the use of animals were performed in accordance with recommendations for the proper care and use of laboratory animals and were performed under authorization through the regulations and policies governing the care and use of laboratory animals (EU directive n° 86/609 and Council of Europe Convention ETS123, EU decree 2001–486 and Statement of Compliance with Standards for Use of Laboratory Animals by Foreign Institutions n° A5388-01, National Institutes of Health (USA).

### Morphometric analysis

Mice were anesthetized with pentobarbital (0.04 mg/kg) and transcardially perfused with saline followed by 80 ml of 4 % paraformaldehyde made in 0.1 M phosphate buffer (pH 7.4). The brains were removed from the skull, and postfixed in the same solution for 24 h. Coronal sections (150 μm), were cut with the aid of a vibratome, and prelabeled with 10^−5^ M 4,6-diamidino-2-phenylindole (DAPI, Sigma D9542).

Cell injection methodology has been described in detail elsewhere [62–64]. Briefly, cells in the posterior third of the left dorsal hippocampus (anteroposterior −2.10 to −2.70 mm from bregma [65] were injected individually with 4 % Lucifer Yellow (CH, Aldrich) in 1 M LiCl (pH 7.4) [66], by passing steady hyperpolarizing current through the electrode (C −0.5 to - 1.0 nA). Current was applied until the distal tips of each neuron fluoresced brightly.

Following injection, the sections were processed with an antibody to Lucifer Yellow (1:400,000 in stock solution [2 % bovine serum albumin (Sigma A3425), 1 % Triton X-100 (BDH 30 632), 5 % sucrose in 0.1 M phosphate buffer]), followed by a biotinylated species specific secondary antibody (Amersham RPN 1004; 1:200 in stock solution) and a biotin-horseradish peroxidase complex (Amersham RPN1051; 1:200 in 0.1 M phosphate buffer). DAB (3,3′-diaminobenzidine; Sigma D 8001) was used as the chromogen. The slides were coded prior to the morphological analysis. The code was not broken until after the quantitative analysis was completed.

For the analysis of dendrite morphometry, neurons were only included in the analysis if they had clearly distinguishable dendritic trees and all dendrites were completely filled. In order to determine whether the dendritic arbor structure is altered in daDREAM mice, neurons were traced with the aid of a computerized data collection system, Neurolucida (Neurolucida V6; MicroBrightfield, Inc., Williston, VT) coupled to an Olympus microscope (BX51), using a 40x objective (NA 0.8) with examination, as needed, at 100x (Oil, NA 1.35). The Sholl analysis [67], which calculates the number of dendritic bifurcations and length at 10 μm-interval distance points starting from the soma, was automatically performed with Neuroexplorer 4.50 program (MicroBrightfield, Inc., Williston, VT). Total dendritic length was also generated.

For the analysis of spine density of granular neurons in the DG, one dendrite with its branches was traced and the dendritic spines were marked. All types of spines were included in the spine counts, and no correction factors were applied to the spine counts, as dendrite tracing at high power allows the visualization of all spines that issue from the dendrites, i.e. the DAB reaction product is more transparent than the Golgi reaction product. The reconstructed data were exported to Neurolucida Explorer (MicroBrightField Inc., Williston, VT) for quantitative analysis. Sholl analysis [67] was applied to determine the spine density at increasing distances from the soma.

For the statistical analysis, spine density was calculated by dividing the number of spines on a segment by the length of the segment and was expressed as the number of spines per 1 μm of dendrite. Densities of spines on dendrites were averaged for a cell mean, and the neurons from each animal were averaged for an animal mean. The total number of spines in the basal dendritic tree of the pyramidal cells in CA1 was calculated by multiplying the mean number of spines of a given portion of dendrite by the mean number of branches for the corresponding region and animal, over the entire dendritic tree (obtained by Sholl analysis) [68]. Normality was tested using the Kolmogorov-Smirnov test. For Sholl analysis, longitudinal distributions were first assessed using 2-way ANOVA considering all interactions, and then, modeled using non-linear regression model fitting to various curves. The best-fitted curves were compared to obtain difference between genotypes. The significance criterion was set at *P* < 0.05. All data shown are presented as mean ± SEM.

### Quantitative real-time PCR

RNA was isolated from hippocampal tissues using TRIzol (InVitrogene), treated with DNAse (Ambion) and reverse transcribed using hexamer primers and Moloney murine leukemia virus reverse transcriptase. To confirm the absence of genomic DNA, each sample was processed in parallel without reverse transcriptase. Quantitative real-time PCR was performed using assays from Applied Biosystems (Additional file 2). The results were normalized by quantification of HPRT mRNA using the specific primers; forward 5′-TTGGATACAGGCCAGACTTTGTT-3′ and reverse 5′-CTGAAGTACTCATTATAGT CAAGGGCATA-3′, and the probe FAM-5′-TTGAAATTCCAGACAAGTTT-3′-MGB.

### Western blot analysis

Mouse hippocampi were quickly removed and one was processed as whole hippocampus and from the other, hippocampal subareas CA1 and DG were dissected as described [69]. Hippocampal tissue was homogenized on ice in NETN buffer (Tris pH 8.0 50 mM, NaCl 250 mM, EDTA 5 mM, NP40 0.5 %, supplemented with protease inhibitors, Complete Roche). Extracts were cleared by centrifugation (14,000 g, 20 min). Samples (15 μg) were analyzed by SDS-PAGE and immunoblot. PVDF membranes were incubated with anti-Arc (C-7, Santa Cruz) and as loading control, with anti-Erk2 (C-14, Santa Cruz). Secondary antibodies used were HRP-conjugated donkey anti-rabbit or -mouse IgG (Jackson; 1 h, room temperature), and signals were detected with ECL Select (GE Healthcare). Band intensity was quantified with QuantityOne software (BioRad).

## Additional files

Additional file 1: Cytoscheletal related genes in daDREAM hippocampus. qPCR analysis. (DOC 84 kb) Additional file 2: Table S1. Assays from Applied Biosystems used for quantitative real-time PCR. (DOC 29 kb)
