# Supplementary material for: Specific cytoarchitectureal changes in hippocampal subareas in daDREAM mice
Source: Mol Brain. 2016 Feb 29;9:22. doi: 10.1186/s13041-016-0204-8 (PMC4772309; doi:10.1186/s13041-016-0204-8)
Supplement: Additional file 1: — Cytoscheletal related genes in daDREAM hippocampus. qPCR analysis. (DOC 84 kb) [file 13041_2016_204_MOESM1_ESM.doc]

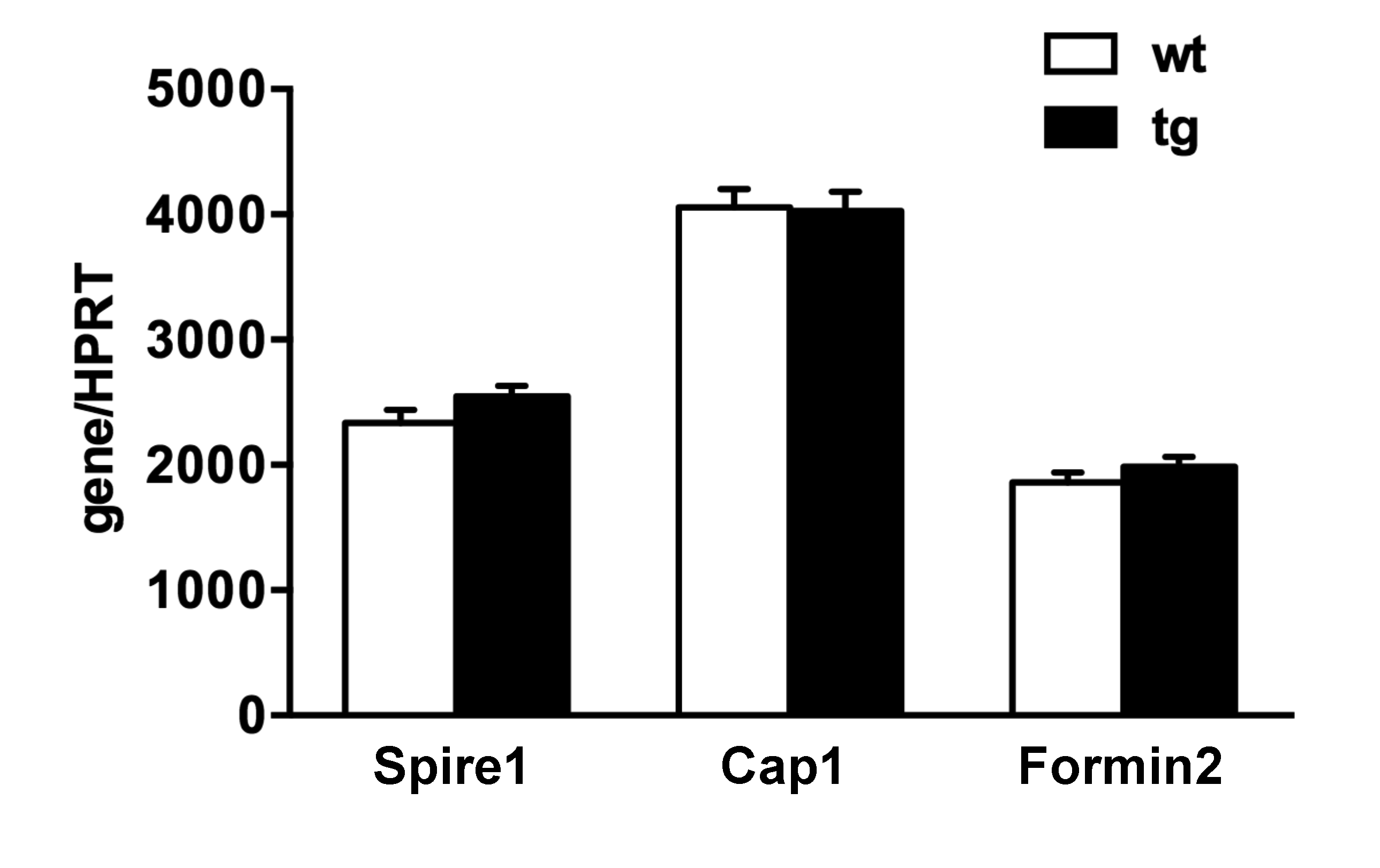


**Figure S1 Cytoskeletal related genes in daDREAM hippocampus.** Quantitative real-time PCR analysis of the indicated genes in hippocampus from wild type (wt) and daDREAM (tg) mice. Values are normalized with respect to HPRT mRNA content.
