# Supplementary material for: Specific cytoarchitectureal changes in hippocampal subareas in daDREAM mice
Source: Mol Brain. 2016 Feb 29;9:22. doi: 10.1186/s13041-016-0204-8 (PMC4772309; doi:10.1186/s13041-016-0204-8)
Supplement: Additional file 2: Table S1. — Assays from Applied Biosystems used for quantitative real-time PCR. (DOC 29 kb) [file 13041_2016_204_MOESM2_ESM.doc]

**Table S1** Assays from Applied Biosystems used for quantitative real-time PCR.

| Arc | Mm00479619_g1 |
| --- | --- |
| Cap1 | Mm00482950_m1 |
| Fhod3 | Mm00614166_m1 |
| Formin1 | Mm00439033_m1 |
| Formin2 | Mm00444598_m1 |
| Gsn | Mm00456679_m1 |
| Spire1 | Mm00813546_m1 |
| Tmod3 | Mm00497523_m1 |
